# Anatomical change during radiotherapy for head and neck cancer, and its effect on delivered dose to the spinal cord

**DOI:** 10.1016/j.radonc.2018.07.009

**Published:** 2019-01

**Authors:** David J. Noble, Ping-Lin Yeap, Shannon Y.K. Seah, Karl Harrison, Leila E.A. Shelley, Marina Romanchikova, Amy M. Bates, Yaolin Zheng, Gillian C. Barnett, Richard J. Benson, Sarah J. Jefferies, Simon J. Thomas, Raj Jena, Neil G. Burnet

**Affiliations:** a Cancer Research UK VoxTox Research Group, University of Cambridge Department of Oncology, Cambridge Biomedical Campus, Addenbrooke’s Hospital, UK; b Oncology Centre, Addenbrooke’s Hospital, Cambridge University Hospitals NHS Foundation Trust, UK; c Cavendish Laboratory, University of Cambridge, UK; d Department of Engineering, University of Cambridge, UK; e Department of Medical Physics and Clinical Engineering, Addenbrooke’s Hospital, Cambridge, UK; f Cambridge Clinical Trials Unit, Box 401, Cambridge University Hospitals NHS Foundation Trust, Addenbrooke’s Hospital, UK; g University of Cambridge School of Clinical Medicine, UK; h Department of Medicine, Cheltenham General Hospital, UK; i University of Manchester, Manchester Academic Health Science Centre and The Christie NHS Foundation Trust, Manchester, UK

**Keywords:** Head & neck neoplasm, Radiotherapy, Intensity-modulated, Radiotherapy, Image-guided, Radiation dosage, Spinal cord, Weight loss

## Abstract

**Background and purpose:**

The impact of weight loss and anatomical change during head and neck (H&N) radiotherapy on spinal cord dosimetry is poorly understood, limiting evidence-based adaptive management strategies.

**Materials and methods:**

133 H&N patients treated with daily mega-voltage CT image-guidance (MVCT-IG) on TomoTherapy, were selected. Elastix software was used to deform planning scan SC contours to MVCT-IG scans, and accumulate dose. Planned (D*_P_*) and delivered (D*_A_*) spinal cord D_2%_ (SCD_2%_) were compared. Univariate relationships between neck irradiation strategy (unilateral vs bilateral), T-stage, N-stage, weight loss, and changes in lateral separation (LND) and CT slice surface area (SSA) at C1 and the superior thyroid notch (TN), and ΔSCD_2%_ [(D*_A_* – D*_P_*) D_2%_] were examined.

**Results:**

The mean value for (D*_A_* – D*_P_*) D_2%_ was −0.07 Gy (95%CI −0.28 to 0.14, range −5.7 Gy to 3.8 Gy), and the mean absolute difference between D_P_ and D*_A_* (independent of difference direction) was 0.9 Gy (95%CI 0.76–1.04 Gy). Neck treatment strategy (*p* = 0.39) and T-stage (*p* = 0.56) did not affect ΔSCD_2%_. Borderline significance (*p* = 0.09) was seen for higher N-stage (N2-3) and higher ΔSCD_2%_. Mean reductions in anatomical metrics were substantial: weight loss 6.8 kg; C1LND 12.9 mm; C1SSA 12.1 cm^2^; TNLND 5.3 mm; TNSSA 11.2 cm^2^, but no relationship between weight loss or anatomical change and ΔSCD_2%_ was observed (all *r*^2^ < 0.1).

**Conclusions:**

Differences between delivered and planned spinal cord D_2%_ are small in patients treated with daily IG. Even patients experiencing substantial weight loss or anatomical change during treatment do not require adaptive replanning for spinal cord safety.

## Highlights

• A cohort of 133 head & neck cancer patients treated with TomoTherapy was examined.
• Differences between planned and delivered maximum spinal cord dose were small.
• Substantial weight loss and anatomical change during treatment was observed.
• No link between weight loss or anatomical change, and dose differences was seen.

Radiotherapy (RT) remains a crucial treatment modality for patients with head and neck cancer (HNC), and IMRT is considered standard of care in most cases [1]. Modern linear accelerators deliver complex dose distribution geometries in 3 dimensions, with plans that include multiple dose levels and simultaneous integrated boosts, whilst respecting dose constraints to key organs at risk (OARs) [2]. Adaptive Radiotherapy (ART) is a logical step in the evolution of external beam X-Ray therapy for HNC. The anatomy of both the patient’s tumour and normal tissues can alter substantially during a course of treatment [3], [4], [5], [6], [7], and these changes may result in differences between intended or *planned* radiation dose to a structure (D*_P_*), and that which is actually *delivered* (D*_A_*) [8]. ART adds a fourth dimension to the complex geometry of an IMRT plan, by amending that geometry during a course of RT to account for these changes [5].

Although the concept of ART is popular, its uptake and utilisation lack uniformity, and it is often performed at the discretion of individual treating physicians [9]. Most work-flows require a new simulation CT scan and a new RT treatment plan, which can be laborious and resource-intensive for the hospital, and onerous for patients [10]. Many clinical protocols are institution-specific, and other centres use this approach only in the research arena. Studies are starting to show both dosimetric [11], and clinical benefit for ART in selected patients [12], but many patients may not require this intervention, and rational selection methods are needed [1], [13].

A crucial dose-limiting OAR for HNC RT is the spinal cord (SC). With modern RT equipment and techniques, severe SC toxicity in the form of transverse myelitis is extremely rare, although Lhermitte’s syndrome remains surprisingly common [14]. Nonetheless, transverse myelitis is catastrophic, and the SC is treated with great respect during planning; conservative dose constraints are given the highest priority in the treatment planning system optimiser. HNC patients experience weight loss and anatomical change during a course of RT treatment [3], [4], and it could be hypothesised that all internal anatomy, including the SC, may be subject to significant differences between D*_P_* and D*_A_* as a result. Available literature suggests that such differences are generally small, and depend on the frequency and quality of image-guidance (IG) [7], [15], [16]. However, these papers study small cohorts, and there are minimal data examining potential associations between weight loss, anatomical change, and differences in SC dose.

The major objectives for this work were: firstly, to examine differences between planned and delivered SC dose in a systematic way in a large cohort; secondly, to measure weight loss and inter-fraction anatomical change in the same patients; finally, to use these data to look for factors that may predict clinically important dose differences, which could in turn be managed by ART strategies.

## Materials and methods

### Patient data and treatment planning

VoxTox is an interdisciplinary research programme based at the University of Cambridge [8], [17], seeking to define differences between D*_P_* and D*_A_*, and better understand relationships between radiation dose and toxicity. The study received ethical approval in February 2013 (13/EE/0008) and is part of the UK Clinical Research Network Study Portfolio (UK CRN ID 13716).

For this pre-planned sub-study, a cohort of 133 HNC patients treated between 2010 and 2016 was defined, with inclusion criteria as follows; squamous or salivary gland carcinomas, a minimum prescription dose of 60 Gy in 30 fractions, neck irradiation to include at least levels II and III unilaterally, and availability of all daily mega-voltage CT (MVCT) images for dose recalculation. Baseline patient characteristics and treatment protocols are summarised in Table 1. All patients in the study were immobilised with a 5-point fixation thermoplastic shell for CT-simulation and treatment. Target and OAR volume definition, as well as CTV and PTV margins, were in-line with a current UK trial protocol [18]. Manual SC contours were expanded axially by 3 mm to a planning organ at risk volume (PRV), to which a dose objective of 46 Gy, and constraint of 50 Gy, was applied. All patients were treated on TomoTherapy Hi-Art units with daily MVCT image guidance (IG) and positional correction with a zero-action level approach (DIPC) [19]. Although IG – MVCTs had a smaller field of view than corresponding kVCT planning scans, all of the upper cervical SC, corresponding to the area of highest cord dose, was imaged daily for all patients. Specifics of the IG workflow used during treatment of patients in this study are detailed in Supplementary material.

**Table 1:** Baseline patient characteristics. (For continuous variables, means and standard deviations are reported, absolute numbers and percentages for proportions).

Characteristic	Number
Age (years)	58.5 (10.1)
Gender	
Male	112 (84.2%)
Female	21 (15.8%)
Baseline weight (kg)	86.0 (18.3)
Disease	
SCC	123 (92.5%)
T0-2	77 (62.6%)
T3-4	46 (37.4%)
N0-1	45 (36.6%)
N2a-3	78 (63.4%)
Oropharynx	84 (68.3%)
Oral cavity	12 (9.8%)
Larynx	11 (8.9%)
Hypopharynx	5 (4.1%)
Nasopharynx	3 (2.4%)
Unknown primary/Other	8 (6.5%)
Salivary gland	10 (7.5%)
Dose/fractionation	
70/35	3 (2.3%)
68/34*	13 (9.8%)
65/30*	88 (66.1%)
60/30	29 (21.8%)
Neck irradiation	
Unilateral	38 (28.6%)
Bilateral	95 (71.4%)
Systemic therapy	
Cisplatin†	75 (56.4%)
Cetuximab†	11 (8.3%)
None	47 (35.3%)

* Primary SCCs of the oro/hypopharynx, and larynx treated with 68 Gy/34# prior to November 2011, 65 Gy/30# thereafter.

† Dose: cisplatin – 40 mg/m^2^ weekly, cetuximab 400 mg/m^2^ loading dose, 240 mg/m^2^ weekly thereafter.

### Computing delivered dose

The planning kVCT scans of all patients were retrieved from archive, tokenised, and reloaded into segmentation software (Prosoma 3.3, MEDCOM, Darmstadt, Germany). To ensure consistency, the SC was manually re-contoured on all planning scans by the first author. The inter-observer consistency of this observer relative to 5 senior radiation oncologists experienced in managing HNC or CNS tumours was found to be acceptable, as previously reported [20]. All MVCT IG imaging (over 4000 MVCT scans), kVCT structure sets, planned dose cubes, and TomoTherapy delivery plans, were transferred to the University of Cambridge Cavendish Laboratory for curation, and automated processing using the Ganga task-management system [17], [21].

Deformable image registration was used to propagate kVCT SC contours onto daily MVCT images. This was performed using the Elastix software [22] – trained and validated as previously described [20]. Daily dose was calculated using a locally implemented ray-tracing algorithm – CheckTomo [8], [20], [23], [24], and voxel dose-histories were accumulated. Final SC delivered dose was reported as a cumulative dose volume histogram (DVH). To minimise sources of discrepancy in the process of dose calculation, planned SC dose was also re-computed using CheckTomo. As the SC is a serial organ [25], we examined maximum dose, and report D_2%_ in line with ICRU 83 [26]. The difference, ΔSCD_2%_, between D*_A_*D_2%_ and D*_P_*D_2%_ was used for comparison with predictive variables. ΔSCD_2%_ is reported as (D*_A_* – D*_P_*), as the clinically relevant difference in this context is higher D*_A_*.

The potential impact of DIPC on delivered SC dose was investigated by simulating D*_A_* values in the absence of any IG. MVCT DICOM headers include details of daily radiographer couch shifts. These values were combined to compute an average couch shift for each patient. The spinal cord contour was translated by the inverse of this shift – relative to the planned dose cube – and D_2%_ recorded in this position. D*_P_*D_2%_ values were then subtracted to give a simulated ‘No IG’ D*_A_*D_2%_ value.

### Predictive variables and anatomical change

To replicate previous methodologies, disease T and N staging data were examined as potential predictors of SC dose differences [27]. Binary classification was used for both metrics (T0-2 vs. T3-4, N0-1 vs. N2-3). Potential differences in SCD_2%_ between patients undergoing unilateral neck irradiation (UNI), and bilateral neck irradiation (BNI), were examined, as was the effect of dose gradient in the vicinity of the spinal cord. To do this, the SC contour on the kVCT scan was grown axially by 6 mm; twice the PRV margin. On the CT slice with the highest SC D*_P_*D_2%_, 4 point doses on this SC + 6 mm ring were measured at 0, 90, 180 and 270 degrees relative to the SC centroid. From these values, corresponding values on the same vector at the edge of the SC contour were subtracted. Totals were summed, then divided by 24 to give a mean dose gradient in Gy/mm (Supplementary Fig. 1).

Weight loss (WL) is a common reason to instigate ART [5], [13], and previous work has directly linked weight loss to changes in SC dose [28]. Patients within VoxTox are weighed at baseline, and weekly during treatment. For this study, baseline weight, and weight measured in the final week of treatment were used to calculate a difference (ΔWL). Twenty-eight patients had missing or inadequate data, leaving 105 patients for this analysis.

**Fig. 1:**
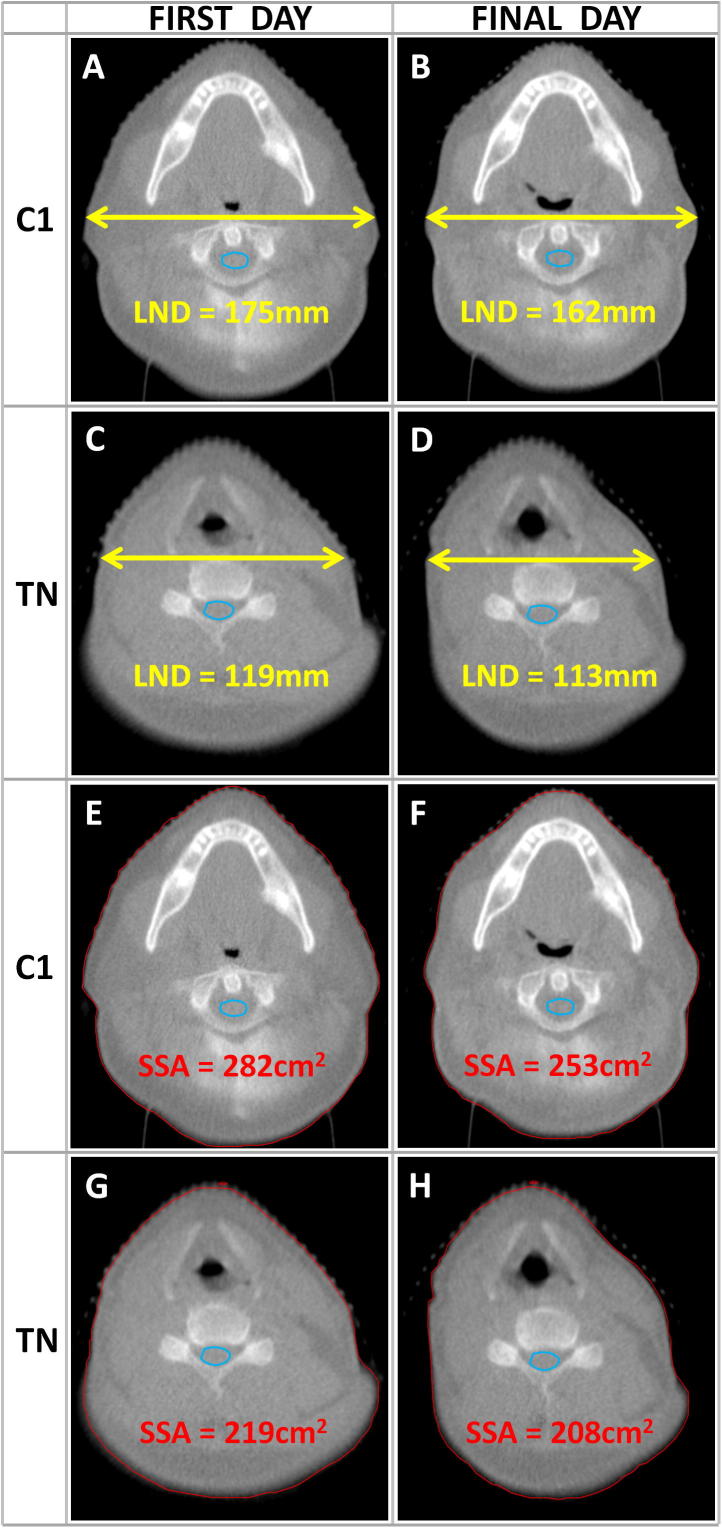
(A–H): Anatomical parameters; lateral neck diameter (LND, captions A–D) and slice surface area (SSA, captions E–H), – measured on the IG-MVCT at the C1 vertebra (C1) and thyroid notch (TN) on the first and final treatment day.

We hypothesised that reducing neck separation might be associated with differences in SCD_2%_. To test this, first and final fraction IG-MVCT images were reloaded into Prosoma. Caliper measurements of lateral neck diameter (LND) at the level of the CI vertebra and superior thyroid notch (TN) were made on both scans (Fig. 1A-D) [27]. Automated external contours were generated on the same CT slice, and the contour (slice) surface area (SSA) was measured. (Fig. 1 E-H) [3]. Changes from the first to the final fraction of RT were recorded as ΔC1LND, ΔC1SSA, ΔTNLND and ΔTNSSA. One patient with very atypical setup (extreme cervical kyphosis; axial plane at C1 included maxillary sinus anteriorly, and spinous process of C3 posteriorly) was excluded, leaving 132 for this analysis.

### Statistical analysis

Patient weight data were directly entered electronically into MOSAIQ data management software (Elekta, Stockholm, Sweden). Anatomical measurement and DVH data were stored in Microsoft Office Excel 2010. Statistical analysis was undertaken using Excel, and R statistical software (R Notebook, R version 3.4.0). Means and 95% confidence intervals are reported for normally distributed data. Links between categorical variables (UNI vs. BNI, T0-2 vs. T3-4, N0-1 vs. N2-3) and ΔSC_D2%_ were analysed with two-sample t-tests; changes in anatomical variables were assessed with paired t-tests. Collinearity between changes in anatomical metrics was assessed with Pearson correlation coefficients (*R*, *R*^2^). Relationships between these changes and ΔSC_D2%_ were examined as univariate relationships with linear regression models (*r*, *r*^2^) [1].

## Results

### Planned versus delivered dose

**Fig. 2:**
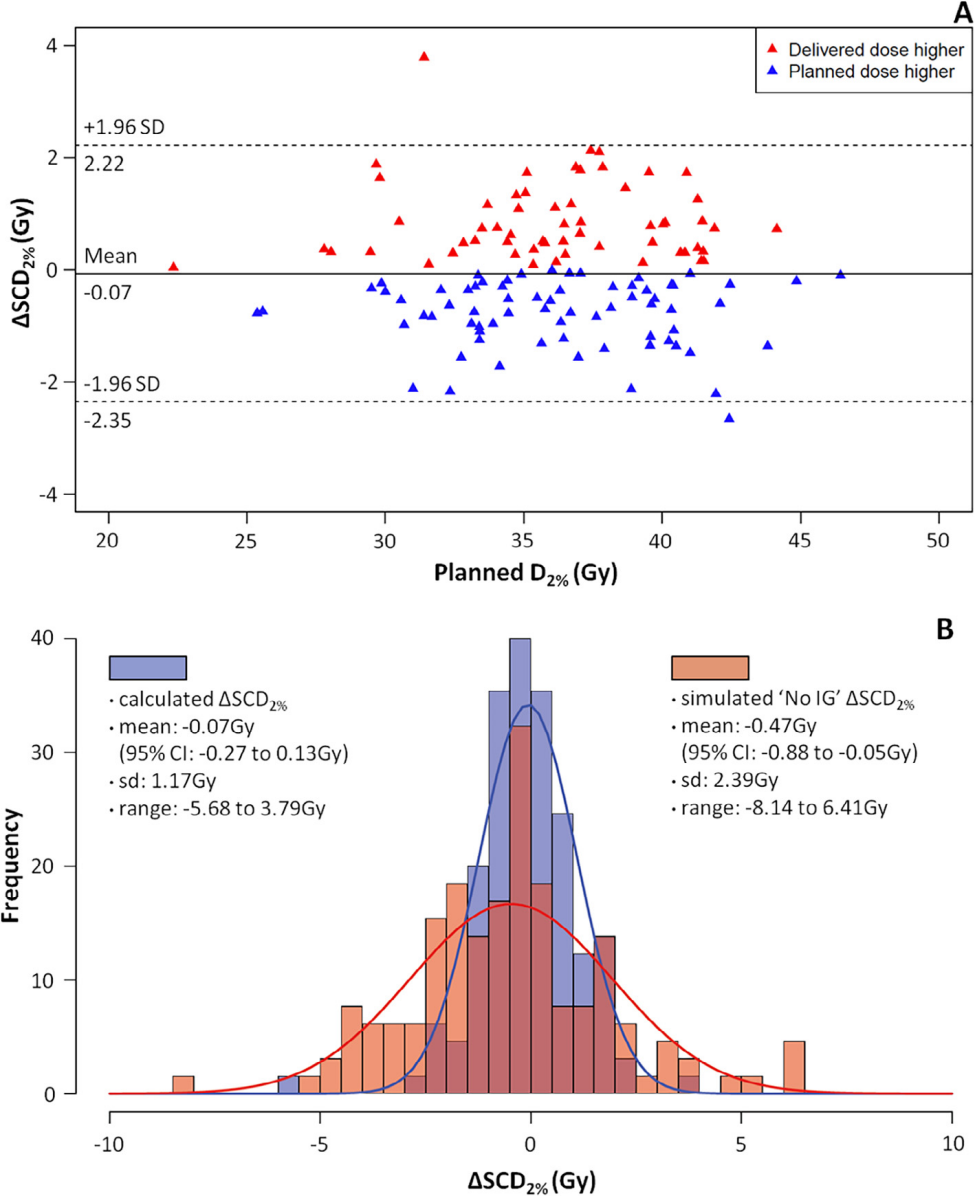
(A and B): Spinal cord dose results (*n* = 133). A – Bland-Altmann plot of delivered – planned D_2% (_ΔSCD_2%_) as a function of planned D_2%_, B – calculated dose differences (blue) and simulated dose differences (red) in the absence of IG plotted as (D*_A_* – D*_P_*)D_2%_ with normal distribution curves.

Mean SCD_2%_ in this cohort was: planned 36.1 Gy (95%CI 35.4 to 36.9, range 22.4–46.4 Gy), delivered 36.1 Gy (95%CI 35.3 to 36.8, range 22.4 to 46.3 Gy). Across all 133 patients, the mean value for (D*_A_* – D*_P_*) D_2%_ was −0.07 Gy (95%CI −0.28 to 0.14, range −5.7 Gy to 3.8 Gy) (Fig. 2A). ΔSCD_2%_ was normally distributed, and the mean difference between D_P_ and D*_A_* (independent of difference direction) was 0.9 Gy (95%CI 0.76 to 1.04 Gy) (Fig. 2B).

Simulated SCD_2%_ in the absence of IG was also normally distributed (Fig. 2B). Interestingly, the sample mean was similar (−0.47 Gy, 95% CI −0.88 to −0.05), but the distribution was substantially broader (mean difference independent of direction 1.8 Gy) and a bigger range was observed, −8.1 Gy to 6.4 Gy.

In 72 (54.1%) patients planned SC dose was higher, whilst in 61 (45.9%) delivered dose was higher. Four patients in the cohort had a delivered D_2%_ that was 2 Gy or more than planned D_2%_, and the biggest observed difference was 3.8 Gy (D*_P_* = 31.4 Gy, D*_A_* = 35.2 Gy). No patient in the cohort had a delivered D_2%_ that breached tolerance dose. There was no relationship between planned D_2%_, and whether or not ΔSCD_2%_ was positive or negative (Fig. 2A).

### Anatomical change

Weight loss, and start-to-end of treatment anatomical change data are shown in Table 2. In order to better understand patterns of anatomical change, and to ensure that univariate relationships between (relative) anatomical metrics and changes in SC dose were independently meaningful, correlation statistics between weight and anatomical change metrics were calculated. Statistical significance (*p* < 0.001) was found for all relationships, but no correlation was sufficiently strong to preclude separate analysis versus dose change. Correlations between weight loss and shape metrics were generally weaker (Pearson’s product moment correlation, *R* 0.28 to 0.40) than relationships between shape metrics themselves (*R* 0.37–0.61). Full results of this analysis are shown in Supplementary Table 1.

**Table 2:** Anatomical change during treatment: weight loss (WL), lateral neck diameter (LND) and slice surface area (SSA). Measurements made at the level of C1 vertebra and superior thyroid notch (TN). *n* = 132 unless otherwise stated.

Metric	Start of treatment (95% CI)	End of treatment (95% CI)	Change (End-Start) (Abs, %) (Range, [%])	Paired *t*-test (*P* value) (95% CI)
Weight (kg) (*n* = 105)	86.0	79.2	−6.8, −7.9	<0.001
	(82.5, 89.6)	(75.9, 82.4)	(−22.1, 6.8)	(−5.8, −8.0)
C1 LND (mm)	154.3	141.4	−12.9, −8.4	<0.001
	(152.1, 156.5)	(139.1, 143.7)	(−22.1, 0.8)	(−11.8, −14.1)
C1 SSA (cm^2^)	225.0	212.9	−12.1, −5.4	<0.001
	(219.7, 230.3)	(208.2, 217.6)	(−15.2, 8)	(−10.5, −13.7)
TN LND (mm)	123.4	118.1	−5.3, −4.3	<0.001
	(118.7, 128.1)	(113.7, 122.6)	(−17.1, 7.5)	(−4.1, −6.5)
TN SSA (cm^2^)	166.9	155.7	−11.2, −6.7	<0.001
	(156.3, 177.5)	(145.7, 165.6)	(−20.7, 13.6)	(−9.2, −13.3)

### Spinal cord dose difference predictors

**Fig. 3:**
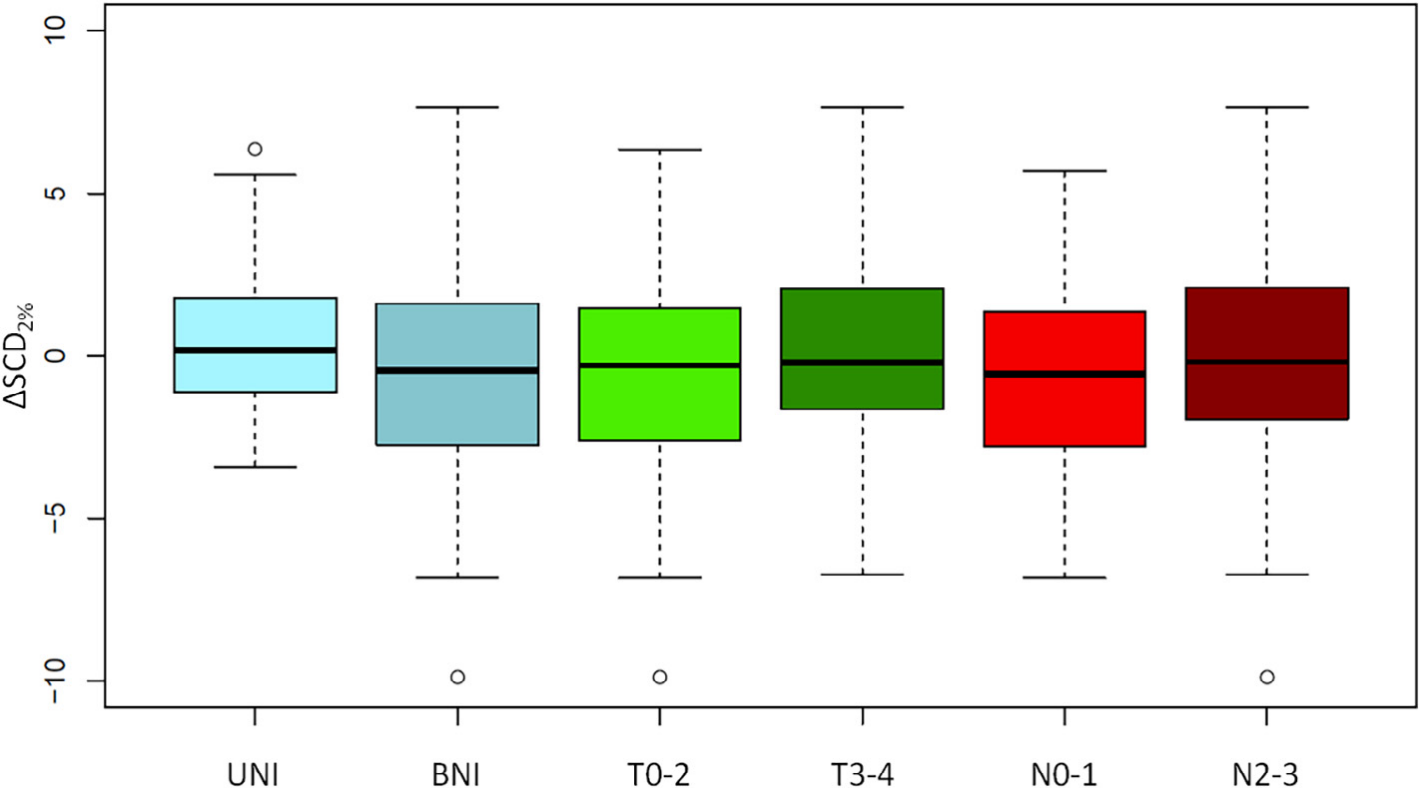
Relationship between neck irradiation strategy (unilateral irradiation [UNI], bilateral irradiation [BNI]), T and N-stage, and changes in spinal cord dose (Delivered D_2%_ – Planned D_2%_ [ΔSCD_2%_]). Relative change in SC dose shown.

Differences in SC dose as a function of neck irradiation strategy, T and N-stage are shown in Fig. 3. Patients undergoing bilateral neck treatment did not have higher SC delivered D_2%_ (mean ΔSCD_2%_ −0.34% for BNI, 0.23% for UNI, 95%CI −0.73 to 1.88%, *p* = 0.39), and higher T-stage did not predict for higher SC dose (mean ΔSCD_2%_ −0.31% for T0-2, 0.04% for T3-4, 95%CI −1.56 to 0.86%, *p* = 0.56). A possible relationship between more advanced neck disease and higher delivered SCD_2%_ was observed (mean ΔSCD_2%_ −0.86% for N0-1, 0.20% for N2-3, 95%CI −2.29 to 0.16%, *p* = 0.088), although this did not reach statistical significance. A relationship between steeper dose gradient in the vicinity of the spinal cord and ΔSCD_2%_ was seen – univariate linear regression *r*^2^ = 0.27 (*p* < 0.001, Supplementary Fig. 2). Mean dose gradient in patients where ΔSCD_2%_ was positive was 0.74 Gy/mm, compared with 0.28 Gy/mm where ΔSCD_2%_ was negative (95%CI for difference in means 0.34–0.57 Gy/mm, *p* < 0.001, two-sample *t*-test).

Univariate linear regression models were used to compare relative change in anatomical metrics, and ΔSCD_2%_. Results are shown in Table 3, and scatter plots are available in Supplementary Fig. 3. In this cohort, no meaningful association between weight loss, or any other metric of shape change was observed (*r*^2^ < 0.05 for all models).

**Table 3:** Univariate linear regression models of weight loss (WL) and shape change (lateral neck diameter – LND, slice surface area – SSA) during treatment against changes in spinal cord dose (SCD_2%_). Anatomical change is measured at the level of the C1 vertebra and superior thyroid notch (TN).

Model	Intercept	B	*r*^2^	*P*
ΔWL vs ΔSCD_2%_	−0.27	0.006	<0.001	0.90
ΔC1LND vs ΔSCD_2%_	0.02	−0.017	<0.001	0.80
ΔC1SSA vs ΔSCD_2%_	0.59	−0.14	0.02	0.08
ΔTNLND vs ΔSCD_2%_	0.13	−0.06	0.002	0.26
ΔTNSSA vs ΔSCD_2%_	−0.12	<0.001	<0.001	0.99

## Discussion

### Spinal cord dose

This study assesses the difference between planned and delivered SC dose in a cohort of 133 patients, compared to sample sizes of 10–20 patients in previously published work [10], [16], [29], [30], [31], [32], [33]. It is the first to do so by accumulating dose from daily IG scans, whilst systematically analysing anatomical change during radiotherapy, and searching for factors that predict for higher than planned delivered dose to the spinal cord.

The magnitude of absolute differences in SC dose seen in this study (0.9 Gy, 2.5% of planned dose) is broadly similar to previously reported data (2.1–4.9%) [10], [29], [30], [31], [32], [33]. However, these studies found SC delivered dose to be systematically higher than planned, in contrast to data presented here. Other authors have not observed such clear systematic differences; Robar and colleagues report a sample mean of 0.3% (sd 4.7%) for ΔD_max 1 cc_, similar to our mean ΔSCD_2%_ of −0.07 Gy (−0.2% of mean D*_P_*D_2%_, sd 3.4%) [6], and a more recent study found a mean SCΔD_max_ of 0.4 Gy (in plans with a 5 mm CTV to PTV margin) [33]. Differences between planned and delivered dose on the TomoTherapy system have also been reported. Using daily MVCT-IG on a cohort of 20 HNC patients undergoing BNI, Duma et al found that 51% of treatment fractions had a D_max_ higher than planned, and an overall difference of 1.2% from the plan [15]. The same authors found a ‘systematic deviation’ between planned and accumulated D_max_ in 75% of patients [16], similar to the 74.4% (99/133) of patients in this study who had a delivered SCD_2%_ >1% different to planned D_2%_.

Nonetheless, the discrepancy between data presented here, and studies in which delivered SC dose is systematically higher, merits further discussion. One possible explanation is the frequency of imaging for dose accumulation. Some researchers have accumulated dose from scheduled (kVCT) rescans, and interpolated dose between timepoints (Castadot – 4 scans, Ahn – 3 scans, Bhide – 4 scans, Cheng – 2 scans) [10], [29], [30], [32], whilst others use weekly CBCT [31]. Interestingly, all these studies reported systematically higher delivered dose. In contrast, authors using daily IG images to accumulate dose saw smaller systematic differences (Duma et al (MVCT), D*_A_* 0.16 Gy higher; van Kranen et al (CBCT), D*_A_* 0.4 Gy) [16], [33].

PRV margins may also be relevant, and reporting on their use is inconsistent. Graff and colleagues did not find greater dose differences for a 4 mm PRV than for the SC itself [34]. However, Castadot et al found that the difference in SC-PRV (4mm margin) Dmax (1.9 Gy) was more than twice that seen for the cord [10], lending credence to the notion that PRV driven optimisation results in steep dose gradients away from the cord itself, and a more homogeneous ‘dose-island’ within. Thus anatomical change and setup error may result in significant differences to PRV dose, without substantial changes to cord dose itself. Our data support this logic; delivered SC dose was systematically higher than planned in patients with a steep dose gradient in the vicinity of the cord itself.

Image guidance policy may also be important. In our simulation, we found mean (direction agnostic) ΔSCD_2%_ to be double the calculated values where daily IG was used (1.8 Gy vs 0.9 Gy). This supports the findings of previous studies, where daily IG use is associated with smaller dose differences [16], [33], and where a direct relationship between frequency of IG, and the magnitude of dose difference is shown [15]. In line with these data, we believe that the small differences seen and reported are due to our policy of DIPC.

### Anatomical change and predictors of dose difference

The data provide no evidence to support the initial hypothesis that patients undergoing bilateral neck treatment would be more likely to see higher delivered SC doses. Furthermore, the results show no effect of disease T-stage on ΔSCD_2%_, in line with previous work [27]. A possible relationship between more advanced nodal disease and higher SC dose is suggested, although statistical significance was not reached. Interestingly, N-stage is an important parameter in models that predict for the need for ART [13].

The observed mean weight loss of 7.9% is similar to previously published figures (5–11.3%) [3], [4], [30], [31], [35], [36]. Crucially, no relationship was seen between weight loss, and higher than planned SC doses, a point on which the literature lacks consensus. The general notion that weight loss leads to significant dosimetric changes is commonly held [1], [37], and one study has shown a link between weight loss and changes in SC dose [28]. Others have not [27], [31], a finding replicated here. Our study is substantially larger than any which has previously addressed this question, and helps to clarify this point.

Patients undergoing radical RT for HNC may undergo shape change independent of weight loss; studies have shown that reducing neck diameter is common during treatment [27], [36], [38]. In-silico modelling suggests reducing neck diameter may lead to higher than planned dose to the SC and brainstem [39], and some clinical data have linked such shape change to higher SC dose. Capelle et al [27] found a significant correlation between reducing LND at the TN and ΔSCD_2%_, although no relationship for reduction at C1. Ahn and colleagues [29] found a significant correlation (*R* = 0.3) between reduction at the level of the ‘mandibular joint’ and increased SC dose, a surprising result given that this structure is superior to the foramen magnum in most patients (in the axial plane). We observed significant reduction in both lateral separation and axial surface area at the level of both the C1 vertebra, and the Thyroid Notch. The shape change data presented here are similar in magnitude to those previously reported [27], [29], [38], but no relationships between these changes and a systematic increase in cord dose were seen. We explain this in 3 ways. Firstly the concept suggested by Graff and colleagues [34], that the spinal cord may be preserved from significant dosimetric change due to its central location, and the use of a PRV margin. This leads to the second point, that dosimetric differences are likely to be random, with minimal impact from systematic error [6]. Finally, most importantly, and based on our simulation of D*_A_* in the absence of IG and the logic of Duma et al [15], we suggest that our policy of DIPC is crucial to the small differences we report.

This is the largest analysis of differences between planned and delivered spinal cord dose in patients undergoing curative radiotherapy for HNC. All patients in the study underwent daily IG with positional correction, and a zero-action level, and observed differences between planned and delivered spinal cord dose were small. No patient had a delivered D_2%_ that breached tolerance dose. Simulated dose differences in the absence of IG were double calculated values, and patients with steep dose gradients in the vicinity of the spinal cord were more likely to have delivered spinal cord dose higher than planned. Weight loss and anatomical change were common and substantial, but had no impact on spinal cord dosimetry. This finding is novel and may assist clinicians making decisions about ART for patients with HNC who undergo significant inter-fraction weight loss and shape change. In patients treated with daily IG, weight loss and shape change does not mandate radiotherapy replanning for spinal cord safety.

## Disclosure and conflict of interest statement

DJN, MR and RJ undertake consultancy work for Microsoft Research.

## Funding

VoxTox received a 5-year programme grant from Cancer Research UK (CRUK) (Ref: C8857/A13405). KH, MR and AMB were supported by the programme grant. DJN is supported by a CRUK Clinical Research Fellowship (Ref: C20/A20917). PLY and SYKS were supported by the Singapore Government. LEAS is supported by the University of Cambridge W D Armstrong Trust Fund. NGB was supported by the NIHR Cambridge Biomedical Research Centre.

## References

[1] Brouwer C.L., Steenbakkers R.J., Langendijk J.A., Sijtsema N.M. (2015). Identifying patients who may benefit from adaptive radiotherapy: Does the literature on anatomic and dosimetric changes in head and neck organs at risk during radiotherapy provide information to help?. Radiother Oncol.

[2] Gregoire V., Jeraj R., Lee J.A., O'Sullivan B. (2012). Radiotherapy for head and neck tumours in 2012 and beyond: conformal, tailored, and adaptive?. Lancet Oncol.

[3] Barker J.L., Garden A.S., Ang K.K., O'Daniel J.C., Wang H., Court L.E. (2004). Quantification of volumetric and geometric changes occurring during fractionated radiotherapy for head-and-neck cancer using an integrated CT/linear accelerator system. Int J Radiat Oncol Biol Phys.

[4] Ottosson S., Zackrisson B., Kjellen E., Nilsson P., Laurell G. (2013). Weight loss in patients with head and neck cancer during and after conventional and accelerated radiotherapy. Acta Oncol.

[5] Hansen E.K., Bucci M.K., Quivey J.M., Weinberg V., Xia P. (2006). Repeat CT imaging and replanning during the course of IMRT for head-and-neck cancer. Int J Radiat Oncol Biol Phys.

[6] Robar J.L., Day A., Clancey J., Kelly R., Yewondwossen M., Hollenhorst H. (2007). Spatial and dosimetric variability of organs at risk in head-and-neck intensity-modulated radiotherapy. Int J Radiat Oncol Biol Phys.

[7] Han C., Chen Y.J., Liu A., Schultheiss T.E., Wong J.Y. (2008). Actual dose variation of parotid glands and spinal cord for nasopharyngeal cancer patients during radiotherapy. Int J Radiat Oncol Biol Phys.

[8] Shelley L.E.A., Scaife J.E., Romanchikova M., Harrison K., Forman J.R., Bates A.M. (2017). Delivered dose can be a better predictor of rectal toxicity than planned dose in prostate radiotherapy. Radiother Oncol.

[9] Gregoire V., Langendijk J.A., Nuyts S. (2015). Advances in Radiotherapy for Head and Neck Cancer. J Clin Oncol.

[10] Castadot P., Geets X., Lee J.A., Gregoire V. (2011). Adaptive functional image-guided IMRT in pharyngo-laryngeal squamous cell carcinoma: is the gain in dose distribution worth the effort?. Radiother Oncol.

[11] Schwartz D.L., Garden A.S., Shah S.J., Chronowski G., Sejpal S., Rosenthal D.I. (2013). Adaptive radiotherapy for head and neck cancer–dosimetric results from a prospective clinical trial. Radiother Oncol.

[12] Chen A.M., Daly M.E., Cui J., Mathai M., Benedict S., Purdy J.A. (2014). Clinical outcomes among patients with head and neck cancer treated by intensity-modulated radiotherapy with and without adaptive replanning. Head Neck.

[13] Brown E., Owen R., Harden F., Mengersen K., Oestreich K., Houghton W. (2015). Predicting the need for adaptive radiotherapy in head and neck cancer. Radiother Oncol.

[14] Laidley H.M., Noble D.J., Barnett G.C., Forman J.R., Bates A.M., Benson R.J. (2018). Identifying risk factors for L'Hermitte's sign after IMRT for head and neck cancer. Radiat Oncol.

[15] Duma M.N., Kampfer S., Schuster T., Aswathanarayana N., Fromm L.S., Molls M. (2012). Do we need daily image-guided radiotherapy by megavoltage computed tomography in head and neck helical tomotherapy? The actual delivered dose to the spinal cord. Int J Radiat Oncol Biol Phys.

[16] Duma M.N., Schuster T., Aswathanarayana N., Fromm L.S., Molls M., Geinitz H. (2013). Localization and quantification of the delivered dose to the spinal cord. Predicting actual delivered dose during daily MVCT image-guided tomotherapy. Strahlenther Onkol.

[17] Burnet N.G.S.J., Romanchikova M., Thomas S.J., Bates A.M., Wong E., Noble D.J. (2017). Applying physical science techniques and CERN technology to an unsolved problem in radiation treatment for cancer: the multidisciplinary 'VoxTox' research programme. CERN IdeaSquare. J Experiment Innov..

[18] Thomson D., Yang H., Baines H., Miles E., Bolton S., West C. (2014). NIMRAD – a phase III trial to investigate the use of nimorazole hypoxia modification with intensity-modulated radiotherapy in head and neck cancer. Clin Oncol (R Coll Radiol).

[19] Burnet N.G., Adams E.J., Fairfoul J., Tudor G.S., Hoole A.C., Routsis D.S. (2010). Practical aspects of implementation of helical tomotherapy for intensity-modulated and image-guided radiotherapy. Clin Oncol (R Coll Radiol).

[20] Yeap P.L., Noble D.J., Harrison K., Bates A.M., Burnet N.G., Jena R. (2017). Automatic contour propagation using deformable image registration to determine delivered dose to spinal cord in head-and-neck cancer radiotherapy. Phys Med Biol.

[21] Moscicki J.T., Brochu F., Ebke J., Egede U., Elmsheuser J., Harrison K. (2009). GANGA: A tool for computational-task management and easy access to Grid resources. Comput Phys Commun.

[22] Klein S., Staring M., Murphy K., Viergever M.A., Pluim J.P.W. (2010). elastix: a toolbox for intensity-based medical image registration. IEEE T Med Imaging.

[23] Thomas S.J., Eyre K.R., Tudor G.S., Fairfoul J. (2012). Dose calculation software for helical tomotherapy, utilizing patient CT data to calculate an independent three-dimensional dose cube. Med Phys.

[24] Thomas S.J., Romanchikova M., Harrison K., Parker M.A., Bates A.M., Scaife J.E. (2016). Recalculation of dose for each fraction of treatment on TomoTherapy. Br J Radiol.

[25] Kirkpatrick J.P., Van-Der-Kogel A.J., Schultheiss T.E. (2010). Radiation dose–volume effects in the spinal cord. Int J Radiation Oncol Biol Phys.

[26] ICRU., Measurements ICoRUa. (2010). Prescribing, recording, and reporting photon-beam intensity-modulated radiation therapy (IMRT). ICRU Report 83. J ICRU.

[27] Capelle L., Mackenzie M., Field C., Parliament M., Ghosh S., Scrimger R. (2012). Adaptive radiotherapy using helical tomotherapy for head and neck cancer in definitive and postoperative settings: initial results. Clin Oncol (R Coll Radiol).

[28] Wang X., Lu J., Xiong X., Zhu G., Ying H., He S. (2010). Anatomic and dosimetric changes during the treatment course of intensity-modulated radiotherapy for locally advanced nasopharyngeal carcinoma. Med Dosim.

[29] Ahn P.H., Chen C.C., Ahn A.I., Hong L., Scripes P.G., Shen J. (2011). Adaptive planning in intensity-modulated radiation therapy for head and neck cancers: single-institution experience and clinical implications. Int J Radiat Oncol Biol Phys.

[30] Bhide S.A., Davies M., Burke K., McNair H.A., Hansen V., Barbachano Y. (2010). Weekly volume and dosimetric changes during chemoradiotherapy with intensity-modulated radiation therapy for head and neck cancer: a prospective observational study. Int J Radiat Oncol Biol Phys.

[31] Ho K.F., Marchant T., Moore C., Webster G., Rowbottom C., Penington H. (2012). Monitoring dosimetric impact of weight loss with kilovoltage (kV) cone beam CT (CBCT) during parotid-sparing IMRT and concurrent chemotherapy. Int J Radiat Oncol Biol Phys.

[32] Cheng H.C., Wu V.W., Ngan R.K., Tang K.W., Chan C.C., Wong K.H. (2012). A prospective study on volumetric and dosimetric changes during intensity-modulated radiotherapy for nasopharyngeal carcinoma patients. Radiother Oncol.

[33] van Kranen S., Hamming-Vrieze O., Wolf A., Damen E., van Herk M., Sonke J.J. (2016). Head and Neck Margin Reduction With Adaptive Radiation Therapy: Robustness of Treatment Plans Against Anatomy Changes. Int J Radiat Oncol Biol Phys.

[34] Graff P., Hu W., Yom S.S., Pouliot J. (2012). Does IGRT ensure target dose coverage of head and neck IMRT patients?. Radiother Oncol.

[35] Langius J.A., Doornaert P., Spreeuwenberg M.D., Langendijk J.A., Leemans C.R., van Bokhorst-de van der Schueren M.A. (2010). Radiotherapy on the neck nodes predicts severe weight loss in patients with early stage laryngeal cancer. Radiother Oncol.

[36] Mazzola R., Ricchetti F., Fiorentino A., Di Paola G., Fersino S., Giaj Levra N. (2016). Cachexia induces head and neck changes in locally advanced oropharyngeal carcinoma during definitive cisplatin and image-guided volumetric-modulated arc radiation therapy. Eur J Clin Nutr.

[37] Brouwer C.L., Steenbakkers R.J., van der Schaaf A., Sopacua C.T., van Dijk L.V., Kierkels R.G. (2016). Selection of head and neck cancer patients for adaptive radiotherapy to decrease xerostomia. Radiother Oncol.

[38] Senkus-Konefka E., Naczk E., Borowska I., Badzio A., Jassem J. (2006). Changes in lateral dimensions of irradiated volume and their impact on the accuracy of dose delivery during radiotherapy for head and neck cancer. Radiother Oncol.

[39] Chen C., Fei Z., Chen L., Bai P., Lin X., Pan J. (2014). Will weight loss cause significant dosimetric changes of target volumes and organs at risk in nasopharyngeal carcinoma treated with intensity-modulated radiation therapy?. Med Dosim.

