# Supplementary material for: Anatomical change during radiotherapy for head and neck cancer, and its effect on delivered dose to the spinal cord
Source: Radiother Oncol. 2019 Jan;130:32–8. doi: 10.1016/j.radonc.2018.07.009 (PMC6358720; doi:10.1016/j.radonc.2018.07.009)
Supplement: Supplementary data 1 [file mmc1.pdf]

Protocol

GV2.05
April 2018

On-treatment volumetric image acquisition and matching protocol

TOMOTHERAPY – HEAD & NECK PROTOCOL

Site	Scan slice width<br>Coarse (C)<br>Normal (N)<br>Fine (F)	Scan slice selection (choose)	Match priority & check coverage	OARs to Review (check)	Actioning (if fail check)		Ref.	Skill Level
					Issue	Resolution		
**Head & Neck**	C	**Whole PTV for 1st & next fraction after sig. change**<br>(Re-planned or sig. weight change seen - corrected by shimming (± 3mm) or un-correctable by shimming)<br>Request DDA to assess dosimetric impact on positional reproducibility/ contour change<br>Avoid lenses unless requested by oncologist<br><br>**Subsequent fractions**<br>Reduce scan volume at radiographer discretion	1. Initial match bony anatomy<br>2. Final match primary soft tissue target<br>3. Check coverage of secondary priority target anatomy using appropriate 95% isodose line	1. Contour change both internal and external<br>2. Brain stem, lenses, spinal cord, optic pathway	• Positional error in pitch/yaw (seen on imaging)<br>• Not possible to match throughout entire volume (target not within planned 95% isodose line<br>• Internal change to OAR	• Reposition patient<br>• Adjust shims as needed<br>• If >3mm shim added under head/ shoulders, request DDA (review before next #)	CP/head & neck	3
					• Internal change to OAR (>95% isodose structure	• Request DDA and / or refer to oncologist		
