# Supplementary material for: Anatomical change during radiotherapy for head and neck cancer, and its effect on delivered dose to the spinal cord
Source: Radiother Oncol. 2019 Jan;130:32–8. doi: 10.1016/j.radonc.2018.07.009 (PMC6358720; doi:10.1016/j.radonc.2018.07.009)

**Supplementary Figure 1 (A-D):** Schematic of method for calculating dose gradient in the vicinity of the spinal cord.

A – Step 1


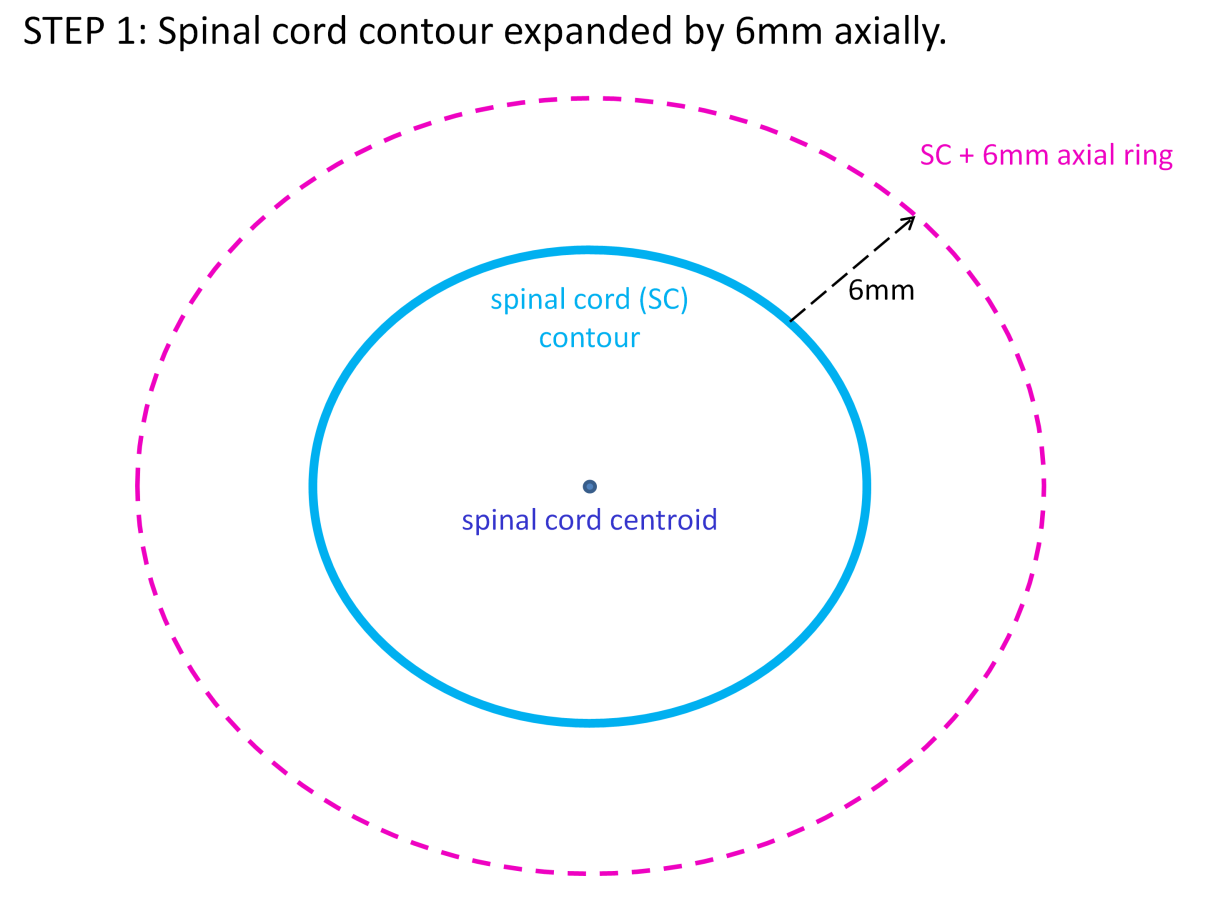


B – Step 2


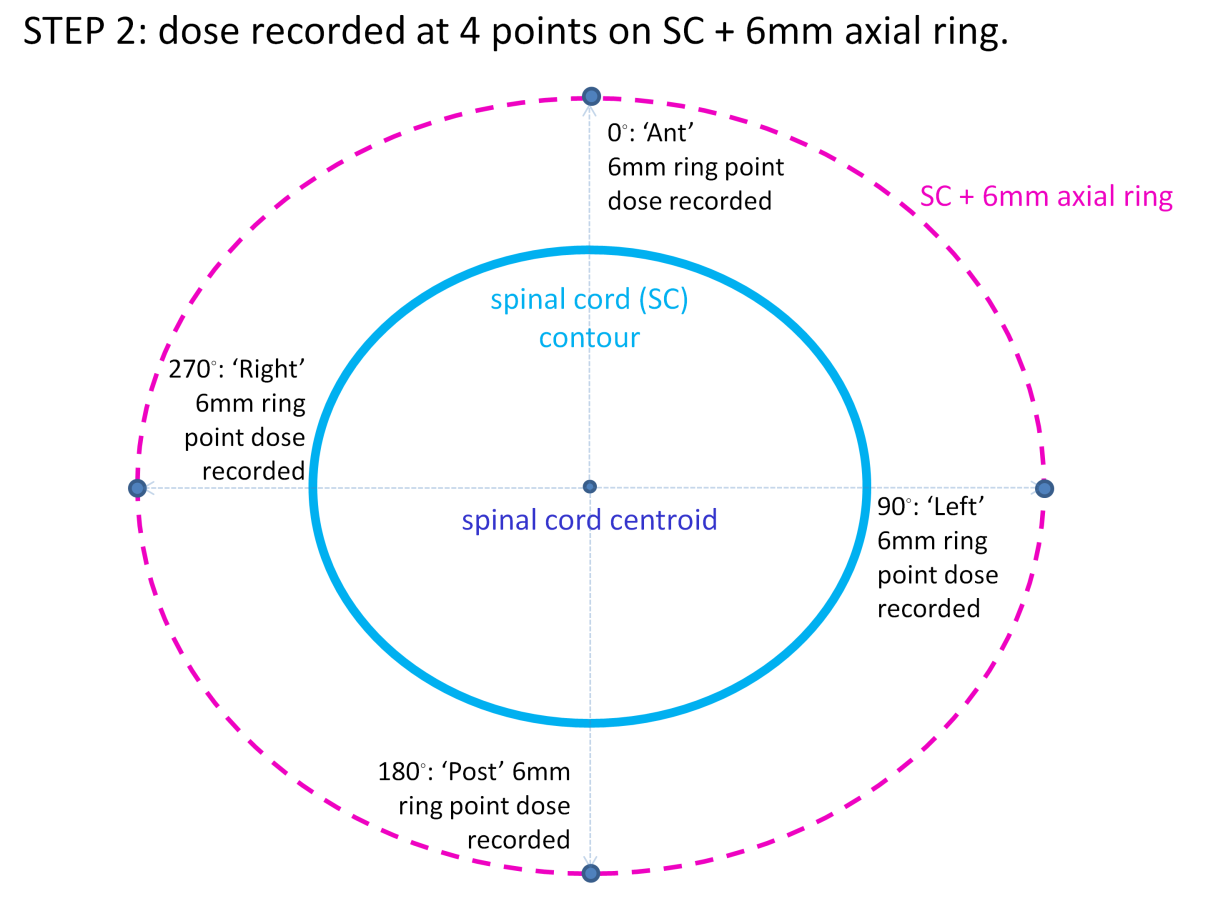


C – Step 3


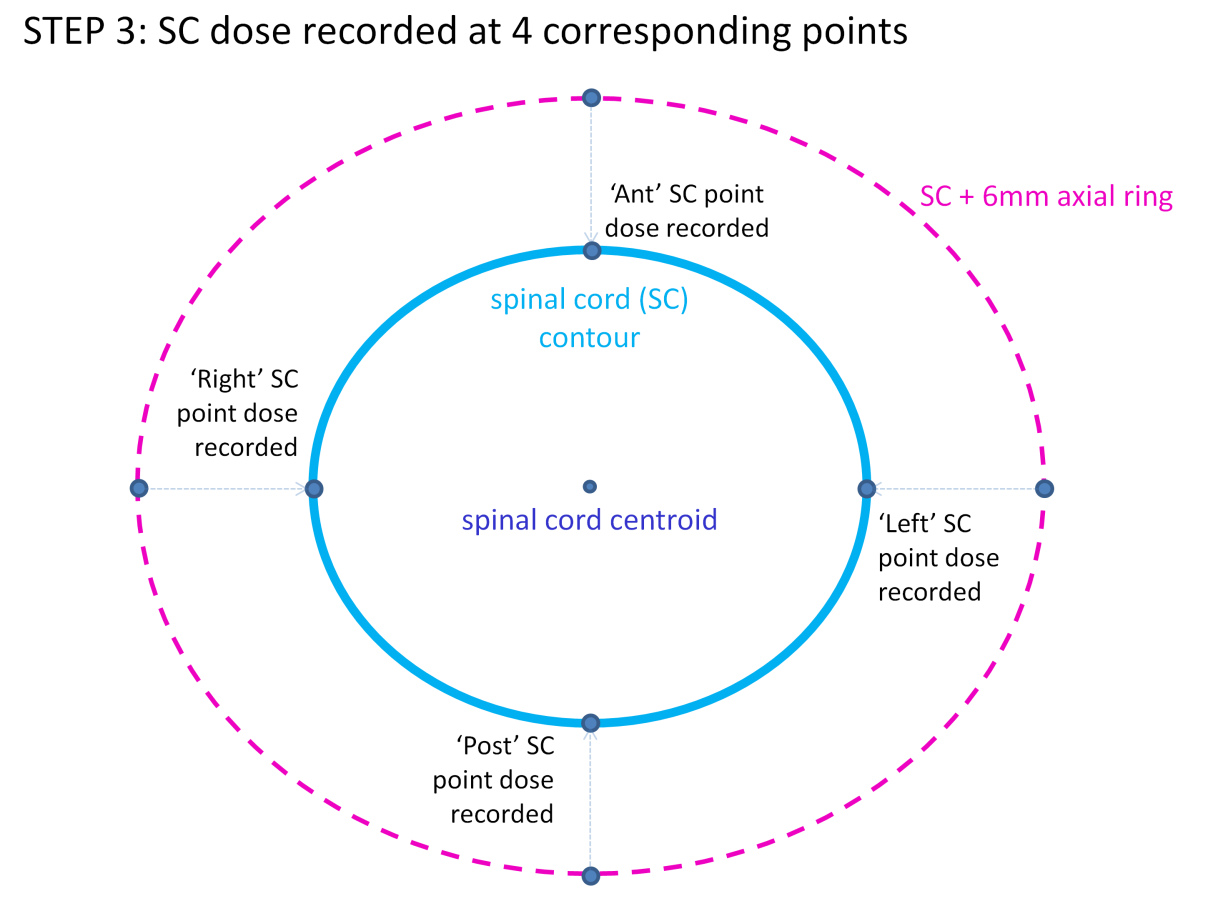


D- Step 4


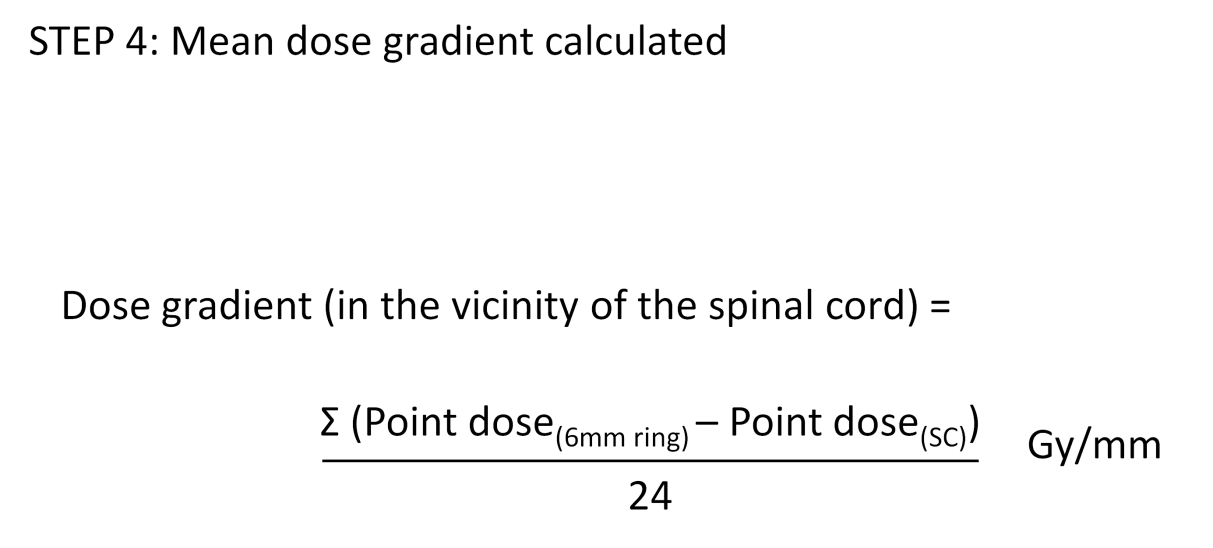

Supplement: Supplementary data 2 [file mmc2.docx]
