# Supplementary material for: Anatomical change during radiotherapy for head and neck cancer, and its effect on delivered dose to the spinal cord
Source: Radiother Oncol. 2019 Jan;130:32–8. doi: 10.1016/j.radonc.2018.07.009 (PMC6358720; doi:10.1016/j.radonc.2018.07.009)
Supplement: Supplementary data 3 [file mmc3.docx]

**Supplementary Figure 2:** Scatter plot of (relative) spinal cord dose differences (ΔSCD_2%_) as a function of dose gradient in the vicinity of the spinal cord.
