# Supplementary material for: Anatomical change during radiotherapy for head and neck cancer, and its effect on delivered dose to the spinal cord
Source: Radiother Oncol. 2019 Jan;130:32–8. doi: 10.1016/j.radonc.2018.07.009 (PMC6358720; doi:10.1016/j.radonc.2018.07.009)
Supplement: Supplementary data 4 [file mmc4.docx]

**Supplementary Figure 3 (A-E):** Scatter plots of univariate relationships between changes in anatomy and spinal cord dose.

A – Weight loss versus ΔSCD_2%_

B – Separation change at C1 versus ΔSCD_2%_

C – Slice surface area change at C1 versus ΔSCD_2%_

D - Separation change at C4 (Thyroid notch) versus ΔSCD_2%_

__

E - Slice surface area change at C4 versus ΔSCD_2%_
