# Supplementary material for: Anatomical change during radiotherapy for head and neck cancer, and its effect on delivered dose to the spinal cord
Source: Radiother Oncol. 2019 Jan;130:32–8. doi: 10.1016/j.radonc.2018.07.009 (PMC6358720; doi:10.1016/j.radonc.2018.07.009)
Supplement: Supplementary data 5 [file mmc5.docx]

**Supplementary Table 1:** Univariate relationships between weight loss during treatment and individual anatomical change metrics (Pearson’s product moment correlation coefficient, R)

| Test | P value | R | 95%CI for R |
| --- | --- | --- | --- |
|  |  |  |  |
| weight loss vs C1 LND | < 0.001 | 0.40 | 0.22 to 0.55 |
| weight loss vs C1 SSA | < 0.001 | 0.36 | 0.19 to 0.52 |
| weight loss vs TN LND | 0.003 | 0.28 | 0.10 to 0.45 |
| weight loss vs TN SSA | < 0.001 | 0.40 | 0.23 to 0.55 |
| C1 LND vs C1 SSA | < 0.001 | 0.50 | 0.36 to 0.62 |
| C1 LND vs TN LND | < 0.001 | 0.37 | 0.22 to 0.51 |
| C1 LND vs TN SSA | < 0.001 | 0.50 | 0.36 to 0.62 |
| C1 SSA vs TN LND | < 0.001 | 0.47 | 0.33 to 0.60 |
| C1 SSA vs TN SSA | < 0.001 | 0.48 | 0.34 to 0.60 |
| C4 LND vs C4 SSA | < 0.001 | 0.61 | 0.49 to 0.71 |
